# *In Vivo* Corrosion of Two Novel Magnesium Alloys ZEK100 and AX30 and Their Mechanical Suitability as Biodegradable Implants

**DOI:** 10.3390/ma4061144

**Published:** 2011-06-21

**Authors:** Tim Andreas Huehnerschulte, Nina Angrisani, Dina Rittershaus, Dirk Bormann, Henning Windhagen, Andrea Meyer-Lindenberg

**Affiliations:** 1 University of Veterinary Medicine Hannover Foundation, Small Animal Clinic, Buenteweg 9, 30559 Hannover, Germany; E-Mails: Nina.Angrisani@tiho-hannover.de (N.A.); Dina.Rittershaus@tiho-hannover.de (D.R.); 2 Institute of Materials Science, Hannover Center for Production Technology, University of Hannover, An der Universität 2, 30823 Garbsen, Germany; E-Mail: Bormann@iw.uni-hannover.de; 3 Department of Orthopedics, Hannover Medical School, Anna-von-Borries Straße 11, 30167 Hannover, Germany; E-Mail: henning.windhagen@annastift.de; 4 Clinic for Small Animal Surgery and Reproduction, Centre of Clinical Veterinary Medicine, Faculty of Veterinary Medicine, Ludwig-Maximilians-Universitaet Muenchen, Veterinaerstr. 13, 80539 Muenchen, Germany; E-Mail: meyer-lindenberg@chir.vetmed.uni-muenchen.de

**Keywords:** biodegradation, magnesium alloy, mechanical stability, animal model, µ computed tomography

## Abstract

In magnesium alloys, the components used modify the alloy properties. For magnesium implants in contact with bone, rare earths alloys are commonly examined. These were shown to have a higher corrosion resistance than other alloys and a high mechanical strength, but their exact composition is hard to predict. Therefore a reduction of their content could be favorable. The alloys ZEK100 and AX30 have a reduced content or contain no rare earths at all. The aim of the study was to investigate their *in vivo* degradation and to assess the suitability of the *in vivo* µCT for the examination of their corrosion. Implants were inserted in rabbit tibiae. Clinical examinations, X-rays and *in vivo* µCT scans were done regularly. Afterwards implants were analyzed with REM, electron dispersive X-ray (EDX), weighing and mechanical testing. The *in vivo* µCT is of great advantage, because it allows a quantification of the corrosion rate and qualitative 3D assessment of the corrosion morphology. The location of the implant has a remarkable effect on the corrosion rate. Due to its mechanical characteristics and its corrosion behavior, ZEK100 was judged to be suitable, while AX30, which displays favorable degradation behavior, has too little mechanical strength for applications in weight bearing bones.

## 1. Introduction

Prior studies, published in the last century, which investigated the *in vivo* degradation of different magnesium alloys, pointed out their potential suitability as biodegradable implants for osteosynthesis. However, in some alloys there were obvious limitations, such as rapid corrosion with accumulation of gas, insufficient mechanical stability, adverse host reactions and toxic effects [1,2,3,4,5]. Due to lacking knowledge of the potential of alloying magnesium, the early researchers experienced difficulties in controlling its corrosion. This is why magnesium lost consideration and why many surgeons preferred the nondegradable V2A steel or titanium, although implants made of these materials had to be explanted after healing [6]. Nowadays, polymeric implants are widely used for osteosynthesis in non-weight bearing bones. They degrade reliably, but are mechanically insufficient for use in weight bearing bones [7]. Magnesium alloys surpass polymeric implants in terms of mechanical stability [8,9] and their corrosion products induce fewer inflammatory reactions [10]. Therefore, recently, magnesium and its alloys again received great interest as materials for degradable metallic implants [8,11,12,13,14,15,16,17,18,19]. In modern magnesium alloys, the alloying elements are used to modify the corrosion properties and the mechanical characteristics of the material so that an implant meets the requirements for its specific application [9,14,15,20]. For implants in contact with bone, the most commonly examined magnesium alloys are magnesium-aluminum-alloys and magnesium-rare earths-alloys [21].

Rare earths were shown to improve corrosion resistance and to increase the stability of magnesium alloys [22], but problems arise from using them as ligands for degradable biomaterials. First of all the commercially available rare earths are a mixture of the elements of the third group of the periodic table of elements plus the lanthanides. The exact composition is therefore hard to predict [18]. Furthermore, it has been shown that, although rare earths are not highly toxic, in high doses they have numerous toxic effects [23,24]. When used as materials for bone implants, their toxic effects on the bone have to be investigated closely, because the clearance from the bone and the bone marrow is known to be very slow [23,24]. Other authors considered the rare earths toxicity to be negligible [14].

The magnesium alloy LAE442 was shown to be of high biomechanical strength and, although it contains rare earths, to have a good biocompatibility [8,17,18]. Other alloys tested so far, such as AZ91, AZ31 and MgCa0.8, were of too little stability or showed a too rapid *in vitro* and *in vivo* degradation [8,17]. However, since rare earths (RE) are possibly toxic, a reduced content of RE in an alloy might be favorable. ZEK100 and AX30 are two novel, magnesium alloys, that have a reduced content of RE (ZEK100) or contain no rare earths at all (AX30). They were shown *in vitro* to be promising [25] as well as of suitable primary stability. Instead of rare earths, like the favorable LAE442 [17], AX30 contains calcium; an element which occurs in the bone in high quantities and improves the corrosion resistance and biocompatibility [18].

In research on magnesium implants, no noninvasive method to measure the true *in vivo* corrosion of implants in hard tissue exists. The conventional methods used so far are all *ex vivo* methods [26]. The *in vivo* microcomputed tomography (µCT) can be used for *in vivo* scans of the experimental animals and therefore may be a suitable tool to assess the *in vivo* corrosion without influencing it.

The aim of this study was to investigate the *in vivo* degradation characteristics of the two new magnesium alloys AX30 and ZEK100 in an animal model and at the same time to assess the suitability of the *in vivo* µCT as a tool to examine the corrosion of magnesium implants.

## 2. Results and Discussion

### 2.1. Euthanasia

All implants could be explanted without causing damage.

### 2.2. Clinical Examinations

In the clinical examinations performed during the follow up period in all animals, minor swellings and wound reactions surrounding the incision could be found for up to ten days. No lamenesses and no signs of pain were documented. One animal, which was an AX30 3 months group animal, showed a minimal subcutaneous accumulation of gas for merely two days directly after the day of the operation procedure.

### 2.3. X-rays

The X-rays showed that all implants, except two, were located correctly within the middle third of the medullary cavity. One of these two implants was from the ZEK100 3 months group and one of the ZEK100 6 months group and they were both located in the proximal third of the medullary cavity.

All results evaluated using a semiquantitative score related to imaging features caused by changes of the implant due to its degradation and to reactions of the bone to the implant degradation or the operation procedure. The scoring results are displayed in Table 1. Generally, for both materials and nearly all time points, the medians calculated were 0. Minimum scores for both materials and all time points were 0, too. AX30 had higher maximum scores than ZEK100 and most of the maximum scores for imaging features displayed a dependence on the implantation time.

**Table 1:** Results of the X-ray scoring of the tibiae during the clinical follow up period.

				Radiologic changes at the site of implantation								Radiologic changes of the bone adjacent to the implant												Radiologic changes of implant		
**material**	**animal group**	**week**		Formation of new bone at the site of implantation				Accumulation of gas				Formation of new bone at the diaphysis				Hyporadiogenity of the medullary cavity adjecent to the implant				Inhomogenous structure of the corticalis				Structure of the implant		
				[a]	[b]	[c]		[a]	[b]	[c]		[a]	[b]	[c]		[a]	[b]	[c]		[a]	[b]	[c]		[a]	[b]	[c]
**ZEK100**	3 and 6 months groups [d]	0		0	**0**	0		0	**0**	0		0	**0**	0		0	**0**	0		0	**0**	0		0	**0**	0
		2		0	**0**	0		0	**0**	0		0	**0**	0		0	**0**	0		0	**0**	0		0	**0**	0
		4		0	**0**	0		0	**0**	0		0	**0**	0		0	**0**	0		0	**0**	0		0	**0**	0
		6		0	**0**	2		0	**0**	0		0	**0**	0		0	**0**	0		0	**0**	0		0	**0**	0
		8		0	**0**	2		0	**0**	0		0	**0**	0		0	**0**	0		0	**0**	0		0	**0**	0
		12		0	**0**	3		0	**0**	0		0	**0**	0		0	**0**	0		0	**0**	0		0	**0**	0
	6 months	16		0	**0**	1		0	**0**	0		0	**0**	1		0	**0**	0		0	**0**	0		0	**0**	3
		20		0	**0**	3		0	**0**	1		0	**0**	0		0	**0**	0		0	**0**	0		0	**1**	3
		24		0	**0**	2		0	**0**	1		0	**0**	0		0	**0**	0		0	**0**	1		0	**2**	3
**AX30**	3 and 6 months groups [d]	0		0	**0**	0		0	**0**	0		0	**0**	0		0	**0**	0		0	**0**	0		0	**0**	0
		2		0	**0**	1		0	**0**	0		0	**0**	1		0	**0**	0		0	**0**	0		0	**0**	0
		4		0	**1**	3		0	**0**	0		0	**0**	0		0	**0**	1		0	**0**	1		0	**0**	0
		6		0	**1**	3		0	**0**	0		0	**0**	1		0	**0**	1		0	**0**	0		0	**0**	1
		8		0	**0**	3		0	**0**	0		0	**0**	1		0	**0**	1		0	**0**	0		0	**0**	1
		12		0	**0**	3		0	**0**	0		0	**0**	2		0	**0**	1		0	**0**	1		0	**0**	1
	6 months	16		0	**0**	3		0	**0**	0		0	**0**	2		0	**0**	1		0	**0**	0		0	**0**	1
		20		0	**0**	3		0	**0**	0		0	**0**	3		0	**0**	1		0	**0**	1		0	**1**	2
		24		0	**0**	3		0	**0**	1		0	**0**	3		0	**0**	1		0	**0**	1		0	**1**	3

[a] Minimum score given a certain time [b] Median of scores given a certain times [c] Maximum score given a certain time [d] X-rays of the 3 and 6 months combined to one median score.

### 2.4. *In Vivo* Micro Computed Tomography

In total 140 *in vivo* µCT scans were performed during this study. Due to artifacts caused by µCT malfunction or movement of the rabbits seven of the scans had to be excluded.

**Figure 1:**
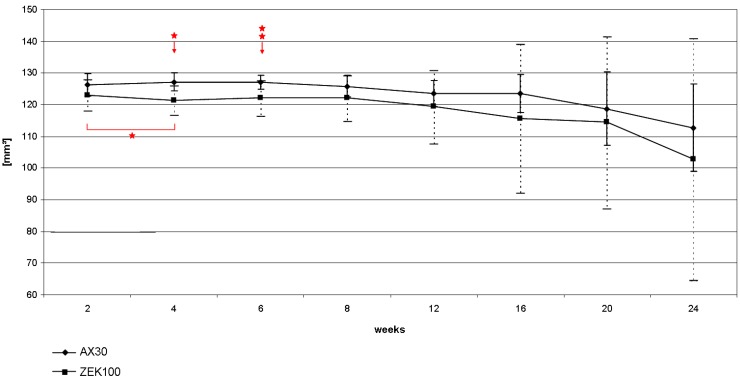
Loss of volume of the implants during implantation measured by the *in vivo* µCT.

In comparison to the scans performed two weeks after implantation, all implants had a reduced volume in the last scan before the sacrifice of the animal (Figure 1). In the AX30 groups the determined implant volume increased in the beginning of the experiment, but not significantly. After the initial increase, the volume of the implants decreased until the end of the experiment and had lost 11.28% compared to week 2. ZEK100 showed a significant initial decrease of the implant volume from week 2 to 4 (–1.29%), but from week 4 to 8 the volume increased again. After week 8 the volume decreased continuously until the end of the experiment. In week 24 the VOI of the ZEK100 implants had lost 16.27% of their volume on average (Figure 1). At all times the average volume of the AX30 implants was higher than that of the ZEK100 implants. At week 4 and week 6 the difference was significant (Figure 1).

The corrosion rates of ZEK100 and AX30 were calculated to be 0.065 mm/y and 0.11 mm/y in the AX30 3 month and 6 months group, respectively. For the ZEK100 3 months group it was 0.067 mm/y, while it was 0.154 mm/y in the ZEK100 6 months group.

**Figure 2:**
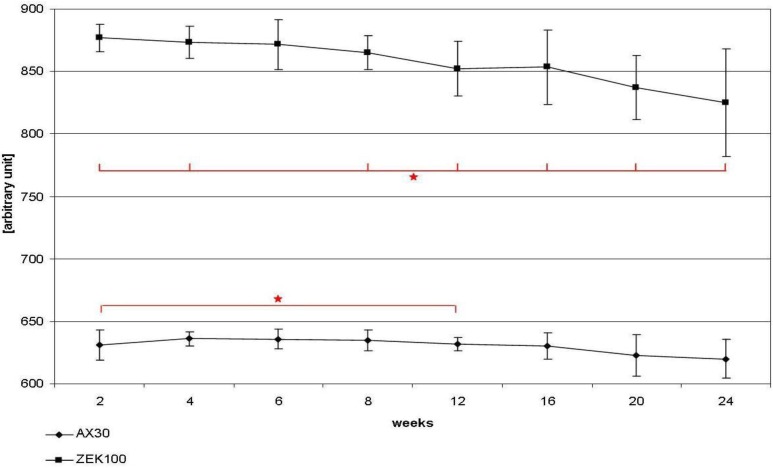
Changes of the apparent density of the implants during implantation measured by the *in vivo* µCT.

The apparent density of the implants is shown in Figure 2. The average apparent density of AX30 implants remained more or less unaltered until week 16. After that the apparent density decreased slightly, but not significantly and lost 1.31% in total. The ZEK100 implants lost apparent density almost continuously. Compared to week 2 the apparent density had decreased significantly from week 8 to week 24. In week 12 the apparent density was reduced by 2.85% on average and in week 24 the reduction was 5.96% on average. Generally the average apparent density of ZEK100 (877.20 ± 11.05 AU) was significantly higher (p < 0.001) at all time points than that of AX30 (631.02 ± 12.05 AU).

**Figure 3:**
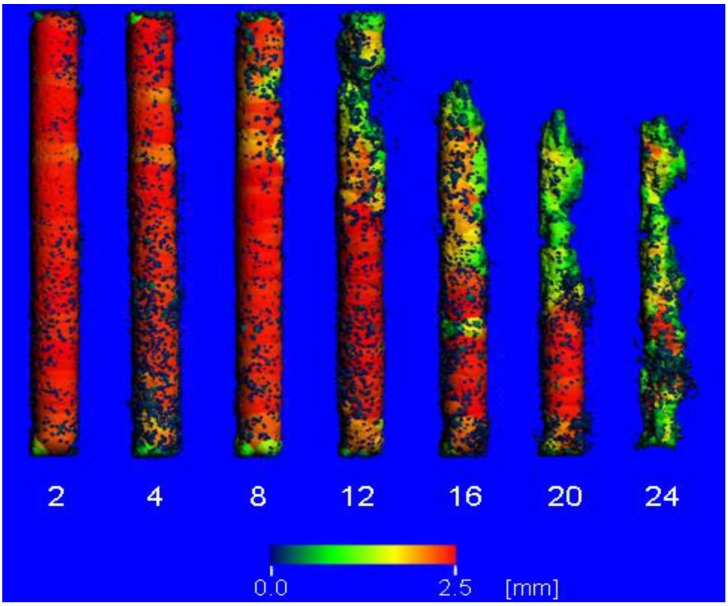
Color mapping of the direct 3D thickness from µCT scans of an exemplary ZEK100 6 months implant. Numbers indicate the respective weeks after implantation.

**Figure 4:**
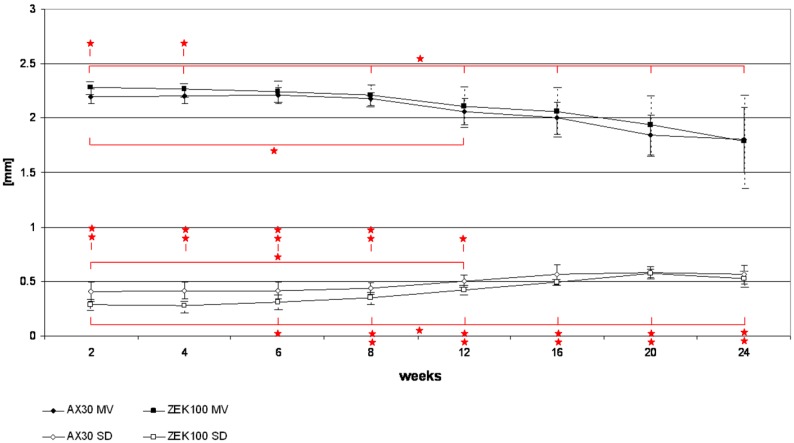
Measurements of the direct 3D thickness of the implants during the implantation measured by the µCT.

Figure 3 shows the course of the corrosion morphology of the ZEK100 6 months implant, which displayed highest degree of corrosion within its group. The implant was one of those that were located in the proximal part of the medullary cavity. It can be seen that the corrosion starts and advances from the proximal end of the implant. In the AX30 group the mean values of the 3D thickness decreased continuously from week 6 on until the end of the experiment. The 3D thickness of the ZEK100 implants decreased continuously from the start of the experiment until the end. In comparison to week 2 the average bin sizes of all other measurements were significantly lower, except for week 6 (Figure 4). During week 2 and 4 the 3D thickness of the AX30 implants was significantly lower than that of the ZEK100 implants (Figure 4). The standard deviation (SD) of the 3D thickness of the ZEK100 implants increased from week 4 to 20 (Figure 4). Compared to week 2 it was higher in week 8, week 12, week 16, week 20 and week 24 (Figure 3). For AX30 only the difference between weeks 2 and 12 was significant (Figure 4). The standard deviation of the 3D thickness of AX30 implants was significantly higher than that of ZEK100 in the scans from weeks 4 to 12 (Figure 4). Afterwards differences were no longer significant.

Fragments of the implant got detached by the corrosion process. (Figure 3) They were very small and found in the vicinity of the implant.

The results of the semiquantitative scoring of the 2D images at the end of the experiments are displayed in Table 2. For both materials the median scores of all parameters were equal or higher in the 6 months groups than in the 3 months groups and the scores given for ZEK100 were equal or higher than those given for AX30, except for the score of endosteal formation of new bone. Gas was found to accumulate intramedullary.

**Table 2:** Resulting score values of the 2D µCT images of the tibiae.

Group	Assessment of the bone structure (cavities)			Bone implant contact (trabeculae)			Endosteal formation of new bone			Periosteal formation of new bone			Accumulation of gas		
n = 5	[a]	[b]	[c]	[a]	[b]	[c]	[a]	[b]	[c]	[a]	[b]	[c]	[a]	[b]	[c]
AX30 3 months	0.00	**0.78**	1.56	0.00	**0.00**	0.00	0.00	**0.00**	0.56	0.00	**0.00**	1.56	0.22	**0.67**	1.10
AX30 6 months	0.56	**1.00**	2.22	0.00	**0.00**	0.00	0.00	**0.33**	0.56	0.00	**1.00**	1.44	0.44	**0.67**	0.78
ZEK100 3 months	0.22	**1.56**	2.67	0.00	**0.00**	0.44	0.00	**0.00**	0.56	1.00	**2.44**	2.78	0.67	**0.89**	2.44
ZEK100 6 months	0.44	**2.00**	3.00	0.00	**0.22**	0.67	0.00	**0.00**	0.11	0.44	**2.44**	2.89	0.78	**1.11**	1.44

[a] Minimum score given [b] Median [c] Maximum score given.

### 2.5. Scanning Electron Microscopy and Energy Dispersive X-ray Analysis

No distinct surface-morphological differences were found between the ZEK100 and the AX30 implants, after the respective implantation times, except for rare earth precipitates in ZEK100 implants.

**Figure 5:**
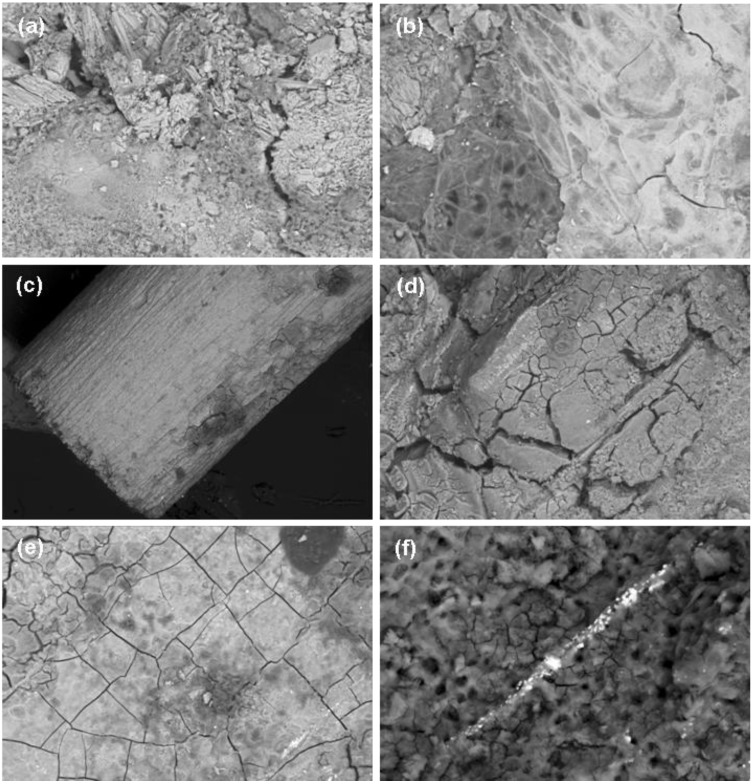
Scanning electron microscopy images of implants after explantation.

The corrosion layers of both materials displayed crystalline needle-like surfaces (Figure 5a) and amorphous structures (Figure 5b), both with high contents of oxygen and also carbon as well as magnesium, shown by EDS analysis. Yet other surfaces were smooth but porous or with fine crack (Figure 5a). They contained phosphorus and calcium in higher quantities although carbon and oxygen were the major elements in their corrosion layer.

After immersion in hydrofluoric acid, the surface morphology of both materials ranged from surfaces with surface textures formed during manufacturing—mainly in the 3 months groups—still preserved (Figure 5c), to surfaces that were rough and flaky layers which displayed multiple fissures and cracks (Figure 5d). In higher magnifications of the surfaces, fissures along the grain boundaries could be found. (Figure 5e) In 1000-fold magnifications pores were found on the surfaces of both alloys. They measured to be approximately 700 to 5000 nm in diameter. In ZEK100 implants precipitates of rare earths were found as elevated streaks, aligned in the extrusion direction (Figure 5f).

### 2.6. Weighing

All remains of the carefully explanted pins were weighed. All implants lost weight during the implantation (Table 3). For the AX30 3 months and the ZEK100 6 months group a significant loss was calculated. After three months AX30 had lost 6.6% of its initial weight, while by that time ZEK100 had already lost 14.1%. After six months AX30 had lost 21.7% and ZEK100 29.0%. The standard deviations of the mean weight loss were high in all groups, except for the AX30 3 months, and they increased time-dependently.

**Table 3:** Weights of the implants initially and after explantation and immersion.

		initial [a]	after [b]	change [c]	[d]
Animal group		[g]	[g]	[%]	p
AX30 3 months (n = 5)	MV	0.214	0.200	**6.6**	0.005
	SD	0.003	0.006		
AX30 6 months (n = 5)	MV	0.214	0.167	21.7	
	SD	0.008	0.043		
ZEK100 3 months (n = 5)	MV	0.211	0.181	14.1	
	SD	0.006	0.021		
ZEK100 6 months (n = 5)	MV	0.211	0.161	**24.0**	0.014
	SD	0.010	0.034		

[a] Average initial weight and standard deviation of the implants in the material-time groups. [b] Average weight and standard deviation after explantation and immersion in hydrofluoric acid. [c] Relative weight loss. [d] Statistical significance.

### 2.7. Mechanical Testing

The results obtained in the 3 point bending tests showed that the initial mechanical stability of the ZEK100 (240.79 N) implants was significantly higher (p = 0.003) than that of the AX30 (177.42 N) implants. The same applied for the mechanical stability after three months (p = 0.001), but not after six months.

All implants lost mechanical stability during the implantation time (Table 4). In the ZEK100 6 months group one implant was degraded to such a great extent that it could not be tested in the experimental setup. In all four groups the maximum force applied to the implant was significantly lower after the implantation than it had been initially (AX30 3 months: −52.33%, AX30 6 months: −70.26%, ZEK100 3 months: −36.58% and ZEK100 6 months: −58.66%). Between the results of the AX30 3 and 6 months groups and the ZEK100 3 and 6 months groups there was no statistically significant difference. When comparing relative losses (Table 4) AX30 had lost significantly more mechanical strength than ZEK100 did (p = 0.033), while after six months the difference was not significant.

**Table 4:** Mechanical properties of the implants measured by 3 point bending after explantation.

	Fmax						ε-Fmax			
		[a]	[b]	[c]	[d]		[a]	[b]	[c]	[d]
		n = 3	n = 5				n = 3	n = 5		
**Animal group**		[N]	[N]	[%]	p		[mm]	[mm]	[%]	p
AX30 3 months	MV	177.4	84.6	**−52.3**	<0.001		5.3	1.0	**−81.8**	0.043
	SD	16.5	7.6				1.6	0.1		
AX30 6 months	MV	177.4	52.8	**−70.3**	<0.001		5.3	0.7	**−87.2**	0.037
	SD	16.5	32.5				1.6	0.3		
ZEK100 3 months	MV	240.8	152.7	**−36.6**	0.003		5.7	4.7	**−16.6**	
	SD	2.4	31.2				0.1	2.1		
ZEK100 6 months	MV	240.8	99.6	**−58.7**	0.006		5.7	2.5	**−55.8**	0.017
	SD	2.4	52.3				0.1	1.3		

[a] Averaged initial maximum force (Fmax) or averaged initial maximum bending at breakage (ε-Fmax); [b] Maximum force after explantation (Fmax) or maximum bending at breakage (ε-Fmax) after explantation; [c] Percentaged losses compared to the initial state. Significant differences are indicated by bold figures of the change; [d] Level of significance.

Results of the measurements of the bending at the moment of breakage (ε-Fmax) are also shown in Table 4. For the AX30 groups the ε-Fmax was significantly lower in the 3 months group (−81.8%) and the 6 months group (−87.2%) than in the initial state of the alloy. In contrast to AX30 the ZEK100 implants did not show a significant decrease of the ε-Fmax after 3 months. But after 6 months (−55.8%) and between months 3 and 6 the difference of the ε-Fmax was significant. Furthermore the ε-Fmax of the AX30 3 months group was significantly lower than that of the ZEK100 3 months group, while the values of the 6 months groups did not differ significantly from each other.

### 2.8. Discussion

The present study was designed to assess the *in vivo* degradation behavior of the two new magnesium alloys ZEK100 and AX30 that were tested *in vitro* to be promising materials [25].

In accordance with other studies, examining the *in vivo* degradation of magnesium implants, the implants made of the two new materials were clinically well tolerated [9,17,18,26]. Yet other studies found only minor clinical changes [16,27] or did not report the clinical progression at all [8,18,29].

Up to now, no noninvasive method to measure the true *in vivo* corrosion of implants in hard tissue without influencing the further corrosion process existed [28]. The conventional methods, which were used in other studies, such as determination of weight or volume loss and measurements of cross sectional areas by radiographic imaging or histology are all *ex vivo* methods [27,28,30]. For the basic methods, like volume or weight measurements, the bone implant complex has to be destroyed. In conventional cross sectional measurements, like histology, the true implant volume had to be extrapolated [28]. Other studies utilized a µCT or Synchrotron-µCT scanner to assess the extent of the corrosion *ex vivo* [16,30,18,27,28]. However, all these methods have in common that they can not identify changes of the corrosion rate *in vivo* and over time in one animal. To our knowledge, this study is the first to use an *in vivo* µCT scanner to determine the true *in vivo* degradation kinetics of implants made of magnesium alloys in hard tissue.

Generally, methods utilizing a CT scanner require a user to define the VOI by manual contouring and determining thresholds, which can lead to methodic errors, because every user is biased [31,32]. In this study the AX30 implants had a higher average volume than they theoretically ought to have by manufacturing. This indicates that either a user dependant error occurred, namely a wrong threshold, or that corrosion products on the surface are not discriminated by the contour. Since the threshold for AX30 was fixed, this error is systemic and applies for all AX30 scans. Relative losses of the average volume of AX30 and ZEK100 implants can therefore still be compared.

In this study the volume of the implants decreased as a result of the degradation, as it was shown in other studies too [16,17,33]. The corrosion mechanism of pure magnesium in aqueous environments is the basis for the corrosion of magnesium alloys [33,34,35]. It is an electrochemical reaction, which forms magnesium hydroxide and hydrogen gas from water and magnesium [33,34]. For implant, the corrosion is an oxygen independent reaction that involves a micro-galvanic coupling between cathodic and anodic areas. It consists of an anodic partial reaction, in which Mg^2+^ and 2e^−^ are formed, and in a cathodic partial reaction, in which 2e^−^ reacts with water to form hydrogen and 2OH^−^. In a subsequent product formation reaction magnesium hydroxide is generated out of the Mg^2+^ and the 2OH^-^ from the anodic and cathodic partial reactions [33,34,35]. Magnesium is prone to special forms of corrosion. First, when exposed to chloride ions, magnesium experiences pitting corrosion, which causes grains to be undercut and to fall out. This is called pitting corrosion. Secondly, filiform corrosion may occur under protective coating or anodized layers. It is caused by a corrosion cell that moves across the surface of the metal [33].

In the study of Krause *et al.* [17], which had an analogous setup of the animal experiment, the water displacement method was used to quantify the loss of volume. In the study of Krause *et al.* after three and six months respectively, LAE442, WE43 and MgCa0.8 had lost more of their initial volume than ZEK100 and AX30 had in the present study [17]. These measurements were done after removal of the corrosion layer [17], which might explain the difference to the results of this study. LAE442, WE43, AZ91 and AZ31 were examined by Witte *et al*. [8], where the corrosion was measured by cross sectional area measurement after 1.5 and 4 months. AZ31, AZ91 and WE43 degraded approximately 3 times faster than LAE442 did [8]. For Mg-Mn-Zn alloys, after 4 months, similar high degradation rates were found [27]. In summary, ZEK100 and AX30 showed a degradation celerity similar to that of LAE442, which was judged to be suitable for an orthopedic implant [17].

Both the ZEK100 3 months group and the AX30 3 months group have a corrosion rate, which is a little higher than that Witte *et al*. [17] published for LAE442 and corrosion rates that are twice as high as the one Zhang *et al.* [36] found for Mg-6Zm *in vivo* corrosion. Since the exact value is known to depend on the implantation time, the differing implantation times have to be kept in mind [17]. Furthermore, it has to be taken into account that different animal models and degradation conditions prevailed [17,26,36].

The average apparent density of the implants measured by *in vivo* µCT declined during the experiment. This study is the first to measure the course of the apparent density and its results are contrary to another publication that considered the apparent density to be constant, although they described a loss of the visual apparent density of implants with preserved shape in *ex vivo* µCT 2D images [18].

In an idealized corrosion process, where no elements are added to the material, no corrosion products precipitate on the surface and no elements are lost from the material, the density of the implant remains constant while the volume reduces [34]. In our study the apparent density clearly changed. A possible explanation could be that the contouring material of the corrosion layer, which had a lower density, was included into the VOI. Among other authors, Song and Atrens [34] reported effects that caused the enrichment of certain alloy components and a depletion of magnesium in the surface film of corroding alloys. It can be assumed that the composition of magnesium alloys changes during degradation and that some ions are eliminated faster than others [35,37]. The apparent density is the averaged density, measured by the µCT, of the uncorroded core of the pin and its surface layers which consist of materials of lower density, such as oxides and hydroxides.

As stated above, a previous study concluded that 2D methods can not be used to reliably extrapolate the corrosion morphology and that for *in vivo* assessments of corrosion morphologies, true 3D methods are needed [26]. The direct 3D determinations of thicknesses of the implants fully fulfill this demand. Magnesium and its alloys have a tendency of pitting corrosion [17,18,33,34], which alters their shape. Consequently, the volume and thereby the average 3D thickness is decreased while its standard deviation is increased.

The initially higher average bin size and the lower standard deviation of the bin sizes of the ZEK100 in comparison to AX30 implants indicate that their initial shape is more uniform than that of the AX30 implants. During the experiment the changes of the average 3D thicknesses and the standard deviations were more or less analogous for both materials. Since the standard deviations of the 3D thicknesses of both materials increased it can be concluded that both alloys experience local corrosion rather than corrosive abrasion of the surface. Furthermore, the results of the measurement of the direct 3D thickness and of the volume measurements support one another. The corrosive formation of distinct pits became visible about the same time (Figure 3) at which the volume measurements revealed an acceleration of the degradation rates. The described alterations in the shape of ZEK100 and AX30 implants could also be shown in the results of the REM analyses.

It showed that the direct 3D measurements can be used to monitor the course of the 3D corrosion morphology *in vivo*, which will be of advantage when the corrosion of implant systems of high complexity needs to be examined. Furthermore they allow an assessment of the intraindividual course of degradation.

As discussed above, the influence of the location of the implant within the medullary cavity on the degradation rate was shown by the volume measurements. The 3D thickness measurements emphasized the effect of the location, because they revealed that the proximally located implant degraded mainly at its proximal end, which was located in the wider part of the medullary cavity (Figure 3).

Studies with a similar experimental setup reported highly variable cross sectional diameters for implants made of MgCa0.8, while the cross sectional diameters for LAE442 were very consistent [17,18,28]. For LAE442 it was concluded that a corrosion layer of little density masks the effect of the corrosion on the shape [18]. Witte *et al.* came to similar results concerning LAE442 [26], while they and others found the remains of AZ31, AZ91, WE43 and Mg-Mn-Zn alloy implants to be of irregular shape [26,27,36].

No other study quantifies the corrosion morphology as it was done in the present study with the direct determination of the 3D thickness, but as ZEK100 und AX30 displayed uneven surfaces their corrosion morphologies are comparable to those of MgCa0.8 [17,18].

The weight loss method is a basic and commonly used method to reliably quantify the degradation of an implant *in vitro* or *in vivo* after explantation [34,38]. However, it has to be taken into account that only a thorough removal of all adherent corrosion products without any damage to the remaining implant gives correct results [34].

In this study all implants lost weight during implantation due to degradation [16,17,33]. Most previous studies determined the reduction of the implant volume by different methods, rather than weight loss [8,17,18,27,39]. The relative losses of weight for ZEK100 and AX30, in this study, can not be compared to changes of the volume of LAE442, WE43 and MgCa0.8, as measured in a previous study [17]. In the present study the extent of the corrosion, as determined by the weight loss method, was greater than that measured volumetrically in the µCT. This might be due to the removal of the corrosion layer before the weighing, but since a decrease of the apparent density was measured in the µCT, the difference in the results of the two methods can be explained.

The statistical analyses of the results obtained by weighing and the volumetric measurements in the µCT revealed high standard deviations due to an inhomogeneous degradation of the implants: The two implants, that were located proximally, exhibited far higher degrees of weight or volume loss than the other implants in their groups and therefore caused the high standard deviations. In literature, different studies reported an effect of the localization on the implant: The bone marrow, which has a good blood supply, makes magnesium implants degrade faster than a poorly perfused environment, like the cortical bone [16,35,37]. In accordance to those findings, in this study the implants, that were located in the wider part of the diaphysis with a lot of surrounding bone marrow, degraded faster than those located in the narrow middle third, which was only a little wider than the implants itself.

Besides the location, factors that may potentially influence the corrosion rate are: An inflammation caused by the operation [3], damage to the implant during the operation, impurities of the implant, microstructural parameters, such as grain size and phase distribution and other individual factors [33].

Since none of the above mentioned factors for an accelerated corrosions rate was found in this study, the above mentioned high standard deviations possibly could have been caused by the differing location of the implants in this study.

All AX30 and ZEK100 implants degraded without a clinically visible accumulation of gas, while gas was commonly found in the 2D images of the µCT scans. The scores showed that the evolution of gas was more distinct in the ZEK100 implants.

It is known that during the corrosion of magnesium and its alloys gaseous hydrogen is generated [8,34] and that hydrogen is eliminated from the bone by diffusion [8]. The authors of previous studies concluded that generally in animals, where no gas accumulates, the rate of the production of hydrogen is lower than its local clearance [8,40]. In this study gas did not become clinically visible, because it did not leave the medullary cavity, but accumulated inside of it.

Other authors, who inserted LAE442 or MgCa0.8 implants intramedullary in rabbit tibiae, also did not find gas clinically and/or in radiographs [17,18], but they did not use an *in vivo* µCT and therefore could not detect a discreet evolution of gas inside of the medullary cavity. In contrast, in another study, in which LAE442, AZ31, AZ91 and WE43 cylinders were implanted in the femoral cavity of guinea pigs, gas bubbles were found clinically and radiographically within the subcutaneous and muscular tissue, because the gas diffused in the surrounding tissue [8].

Although the mechanical characteristics are of utmost importance for fracture fixation in weight bearing bones, most *in vivo* studies focus on the biocompatibility of the alloys rather than on changes of their mechanical characteristics [17,20]. The tensile strength is regarded to be the critical mechanical characteristic of an implant that is supposed to stabilize bone fragments [30], while its ductility is of less importance [17,20]. As it is known, magnesium alloys have a tendency for pitting corrosion [33,34]. Pits locally decrease the cross sectional diameter, which cause stress peaks to accumulate and the implant to fail if they exceed the local mechanical strength [17,41]. It was shown that LAE442 is of high initial tensile strength and that it can be used as material for degradable implants in weight bearing bones [17,18]. The initial tensile strength of ZEK100 is a little lower than that of LAE442. After three months the average mechanical strength of ZEK100 was similar to that of LAE442 (153.2 ± 18.5 N), but after that ZEK100 lost strength much faster (LAE442 after 6 months 134.7 ± 14.7 N) [17]. Since it was shown that LAE442 is of sufficient tensile strength for the use in weight bearing bones initially as well as after three months [17], the same applies for ZEK100. Prior studies pointed out that an initially slow degradation, followed by an acceleration of the corrosion rate once the fracture has healed, would be ideal [17]. This demand has to be enlarged to the mechanical stability. During the healing of a fracture a mechanically fully functional implant is supposed to keep the fragments in tight contact with only little movement. [30,42] This can only be achieved, if a degradable implant sustains an adequate mechanical strength until the time at which an external callus of woven bone, bridging the fragments, has formed [30,43]. In humans, depending on the type of fracture and the age of the person, this may take from one to four months [43]. After its mineralization, the callus needs mechanical induction of the remodeling process, which shapes it [30,42]. An ideal degradable implant should lose stiffness and allow the callus to bear weight. After three months of implantation ZEK100 has the same tensile strength (152.7 N) as LAE442 does [17]. Another alloy, MgCa0.8, which showed an almost linear decrease of its tensile strength and pitting corrosion, proved unsuitable for the use in weight bearing bone [17]. The initial mechanical strength of AX30 is the same as that of MgCa0.8, but AX30 lost mechanical strength even faster [17]. Therefore AX30 seems to be of insufficient strength for uses in weight bearing bones.

As it was shown for other alloys, too [17], the ductility of both materials used in this study was significantly lower after explantation than initially. This was expected because corrosion is known to form fissures and pores on the surface of the magnesium implants [17,39] and because an increased surface roughness leads to a brittle fracture behavior. After six months of implantation, ZEK100 (2.5 mm elongation) had, in an identical setup, a ductility comparable to that of LAE442 (2.56 mm elongation) [17,41]. The scanning electron microscope (SEM) analyses of the implants support this, because they showed that both materials displayed an increased surfaces roughness. For implants that are supposed to stabilize fractures, ductility is of less importance than tensile strength [17], but for other uses ductility could be of greater importance.

The bone reactions to the implant were assessed by a scoring of radiographs and 2D µCT images. The superposition of the detected anatomical structures and the lower resolution radiographs provide a quick overview, but the reactions of the bone to the implant, for example trabecular bone implant contact, can be assessed more precisely by the µCT. The score was based on a previously published scoring, but modified [18,28]. Histology is the method of choice to examine host reactions [44,45] and it is therefore frequently used. The *in vivo* µCT allows an evaluation of the osseous reactions to the implant during the ongoing experiment.

In the 2D µCT images, the 6 months groups of both materials had higher scores for changes of the overall bone structure than the 3 months groups, which can be attributed to the fact that the ongoing corrosion induces reactions of the bone. Furthermore, after 3 and 6 months, the median scores of the ZEK100 implants were twice as high as the score of AX30 implants. As discussed earlier, measurements assessing the extent of corrosion showed that ZEK100 implants had a higher extent of corrosion than those made for AX30. As ZEK100 induces more formation of new bone periosteally than AX30, a relation between osseous changes and the extent of the corrosion can be supposed. The scores for endosteal and trabecular formation of new bone were indifferent for both materials.

Since scoring was modified and the duration of the experiments were different a direct comparison to the studies of Thomann *et al.* [18,28] and the present is not possible. MgCa0.8 and MgCa0.8 with an additional fluoride coating were examined in the same experimental setup, revealed a higher degree of bone formation, especially endosteally and around the implant, which might be assigned to a better biocompatibility [28].

A complete and adequate examination of the biocompatibility has to be done by histology and should be the subject of another study.

## 3. Experimental Section

### 3.1. Implant Material

The two magnesium alloys used in this study are not commercially available. ZEK100 consists of magnesium with 1 wt% of zinc, less than 1 wt% of zirconium and less than 1 wt% of rare earths as alloying elements. AX30 consists of magnesium with 3 wt% of aluminum and less than 1 wt% of calcium. Both alloys were named in accordance with the ASTM standard B275-90 [46].

The manufacture of the ZEK100 and AX30 billets was done in a gravity die-casting process. Due to the high reactivity of liquid magnesium, the material was melted and cast in a protective Argon atmosphere, which was achieved by dynamically circulating Argon around the crucible with a volumetric flow rate of 3 L/min. Both alloys were melted at a temperature of 760 °C. The die used for the casting was heated to 600 °C for ZEK100 and to 560 °C for AX30. The billets were further processed by direct extrusion. Therefore their diameter was reduced to 120 mm by turning on a lathe. Following this, the billets of both materials were soaked at 350 °C in a furnace for 2 h. The extrusion die (orifice diameter of 30 mm) and its recipient were heated to a temperature of 380 °C for ZEK100 and to 400 °C for AX30. The billet was then inserted into and pressed through the die at a ram speed of 1 mm/s for ZEK100 and 1.5 mm/s for AX30. The final implants were 2.5 mm in diameter and 25 mm in length.

All implants were washed in acetone and distilled water in an ultrasonic bath and then separately packed. They were sterilized with gamma radiation at 25 kGy for 8 hours by a commercial provider (BBF Sterilisationsservice, Kernen, Germany) [8,12].

### 3.2. Animal Model and Study Design

The animal experiments carried out in this study were in accordance with a protocol approved by the ethic committee in charge as well as with § 8 of the German Animal Welfare Act. They were legitimized by the Office for Consumer Protection and Food Safety under the approval number 33.9-42502-04-07/1363. For the animal experiment 24 female, adult New Zealand White Rabbits (Charles River, Kisslegg, Germany) with a body weight of 3.5 to 4.5 kg were used. The rabbits were housed in separate cages in a controlled environment. The animals were randomized into four groups of six animals each differing in time and/or material (AX30 3 months, AX30 6 months, ZEK100 3 months and ZEK100 6 months). Within each group there was one animal without an implant, which served as negative control, resulting in 20 implants in total and two negative controls for three months as well as two for six months.

Before the operation procedure all rabbits received subcutaneous injections of meloxicam (0.15 mg kg^−1^, Metacam®, Boehringer Ingelheim, Ingelheim, Germany) and enrofloxacin (10 mg kg^−1^, Baytril® 2.5%, Bayer HealthCare, Leverkuesen, Germany). This medication was continued orally during the following ten days post operatively. To induce anesthesia, the rabbits received intramuscular injections of s-ketaminehydrochloride (10 mg kg^−1^, CP-Pharma, Burgdorf, Germany) and medetomidine (0.125 mg kg^−1^, Domitor®, Pfizer GmbH, Berlin, Germany). After endotracheal intubation, the anesthesia was continued by administering a mixture of isoflurane and oxygen (2 to 3 vol% isoflurane, oxygen airflow 0.4 to 0.6 L/min, Isoba®, Essex Pharma GmbH, Munich, Germany) under spontaneous respiration. Furthermore, the rabbits received an infusion of Paediafusin© (10 mL kg^−1^ h^−1^, Baxter, Unterschleissheim, Germany). Both hind legs were clipped and disinfected. The rabbits were brought to the operation theatre and placed on a heating pad. Shortly before the incision fentanyldihydrogencitrate (10 µg kg^−1^, Fentanyl-Janssen®, Janssen-Cilag GmbH, Neuss, Germany) was given intravenously and from that time the rabbits were ventilated artificially if necessary. On both hind legs an incision of the skin and the fascia underneath was made on the medial side of the tibia, just mediodistal of the tibial tuberosity. After the periosteum had been detached from the tibia, a 2.5 mm wide hole was drilled through the cortex, so that the implant could be placed in the middle third of the medullary cavity using a sterile plastic push. The soft tissue layers were closed separately. After the operation the position of the implants was controlled radiographically in two projections. In the control animals the operation procedure was performed as described above, including the insertion of the push, except for no implant was inserted.

During the follow up the animals were examined clinically on a daily basis. Special attention was paid to the occurrence of pain, lameness and subcutaneous accumulation of gas. X-rays and *in vivo* micro computed tomography (µCT) scans were done every two weeks for the first three months in all of the four groups. In the two 6 months groups scans were performed every four weeks from three months on until the end of the experiment. For the *in vivo* µCT scans the rabbits were anesthetized as described above, however without analgesia.

### 3.3. X-rays

During follow up latero–lateral and anterior–posterior X-rays of the hind legs were performed at 48 kV and 6.3 mAs using a conventional digital system. The X-rays were analyzed using a semiquantitative scoring system (Table 5) with values between 0 and 3 for defined imaging features. The implants were supposed to be positioned in the middle third of the tibial medullary cavity.

**Table 5:** Semiquantitative score for the evaluation of radiographs of the tibiae during follow up.

Radiologic changes at the site of implantation		Radiologic changes of the bone adjacent to the implantation			Radiologic changes of the implant	Score
Formation of new bone at implantation site	Accumulation of gas	Formation of new bone at diaphyses	Hyporadiodensity adjacent to the implant	Inhomogenous cortical structure	Structure of impant	
none	none	none	none	none	as on day of implantation	0
<2 mm width <5 mm length	single bubbles <2 × 4 mm	<1 mm per side	minimal	minimal	minimal structure loss, shape conserved	1
2–4 mm width 5–10 mm length	bubbles (2–4) × (4–8) mm	1–2 mm per side	moderate	moderate	moderate structure loss, shape conserved	2
>4 mm width >2 mm width & >10 mm length	many bubbles >4 × 8 mm	>2 mm per side	severe	severe	severe structure loss, shape altered	3

### 3.4. *In Vivo* Micro Computed Tomography

For the regular *in vivo* scans of the tibiae of the rabbits during the follow up period a micro computed tomography scanner (XtremeCT, Scanco Medical, Zurich, Switzerland) was used. It has a maximum resolution of 41 µm and a maximum image matrix of 3072 × 3072 pixels. The scanner uses a 2D and true 3D multi-planar reformatting evaluation and visualization software, which allows volume registration and 2D and 3D density measurements of user defined regions of interest. 3D analyzes scripts allow further processing of irregularly shaped three-dimensional volumes of interest (VOI). For the scans the anesthetized rabbits were placed in dorsal position within a carbon tube that fitted into the gantry of the µCT. First topograms of the legs were made, in which the implants could be identified. Then the area for the tomogram was determined. The slice thickness was 41 µm and the integration time used was 100 msec per slice. The electron energy used was 60 kVp and the intensity was 900 µA. For each slice the scanner did 1000 projections at 180°. The parameters to be analyzed were: volume, apparent density and 3D-thickness of the implant. For the evaluation the implant was manually contoured in the 2D slices. A threshold, specific for the two different alloys (ZEK100 threshold 127 and AX30 threshold 115), was determined and used for all evaluations. This procedure defined a three-dimensional VOI, which could be further evaluated.

In order to assess the extent of the degradation of the implants, volumetric and apparent density measurements of the VOIs were done. As the µCT was calibrated with a phantom for density measurements, the apparent density is stated in arbitrary units (AU). To further quantify the corrosion rate and the corrosion morphology direct 3D determinations of thickness of the VOIs were performed. Therefore, the thickness was calculated by filling the structure with overlapping spheres of maximal diameter. The diameter of the spheres at each location resembles the local thickness and the average thickness was determined by averaging over the whole structure. This led to histograms of bin sizes with an average 3D thickness and a standard deviation for each implant. A low average bin size with a low standard deviation indicates a high degree of uniform corrosion. A high standard deviation of the histogram is caused by an irregular shape of the remaining implant and therefore it is an indicator for the extent of pitting corrosion.

A semiquantitative scoring system, modified after Thomann *et al.* [18], was used to examine changes of the bone at the end of the experiment. Per animal nine images, evenly distributed over the slices, in which the implant was visible, were scored. The scoring (Table 6) allocated values between 0 and 3 for defined features in the 2D images of the µCT scans. Images without any changes in the osseous structure resulted in a score of 0, while bones with very distinct changes were scored 3.

The *in vivo* corrosion was calculated from the loss of volume according with the following equation proposed by Witte *et al*. [17]: $CR=\frac{\text{Δ}V}{A\cdot t}$. The reduction of the volume (ΔV) is divided by the time (*t*), and area exposed (*A*). The area exposed was calculated to be 206.17 mm² for all implants. The time was calculated per year.

### 3.5. Euthanasia

After the last *in vivo* µCT scan, the anesthetized animals were euthanized by intracardiac injections of pentobarbital (230 mg kg^−1^, Narkodorm®, CP-Pharma, Burgdorf, Germany). Using a handheld circular saw their explanted right tibiae were cut perpendicularly to their long axis 1 cm proximal and distal of the implant and then split longitudinally. The remains of the implant were carefully collected from the medullary cavity and securely stored in a dry atmosphere until further analysis. The left tibiae were collected and securely stored for further investigations in a separate study.

### 3.6. Immersion in Hydrofluoric Acid

The implants were analyzed in a scanning electron microscope (SEM) before and after immersion in hydrofluoric acid (40%) (HA) for five minutes. The immersion in HA removed the corrosion layer and adherent organic material, but did not attack the magnesium. [47] After immersion the implants were rinsed with distilled water and ethanol and left to dry at ambient air. Additionally the implants were weighed in their initial state and after immersion. Mechanical testing was also done after the immersion in HA.

**Table 6:** Semiquantitative score for the osseous reaction to the implant in µCT 2D images of the tibiae.

Feature	Parameter	Score
Overall assessment of the bone structure (cavities)	regular	0
	minor irregularities (<30% of the area)	1
	distinct irregularities (30–60% of the area)	2
	severe irregularities (<60% of the area)	3
Bone implant contact (trabeculae)	none	0
	<1/3 of the implant surface	1
	1/3–2/3 of the implant surface	2
	>2/3 of the implant surface	3
Endosteal formation of new bone	none	0
	<1/3 of the endosteal surface	1
	1/3–2/3 of the endosteal surface	2
	>2/3 of the endosteal surface	3
Periosteal formation of new bone	none	0
	<1/3 of the periosteal surface	1
	and <1/3 of the cortical thickness	
	1/3–2/3 of the periosteal surface	2
	or 1/3–2/3 of the cortical thickness	
	>2/3 of the periosteal surface	3
	or >2/3 of the cortical thickness	
		
Accumulation of gas	none	0
	<1/3 of medullary cavity filled with gas bubbles	1
	1/3–2/3 of medullary cavity filled with gas bubbles	2
	>2/3 of medullary cavity filled with gas bubbles	3

### 3.7. Scanning Electron Microscopy and Energy Dispersive X-ray Analysis

Each implant was analyzed in a scanning electron microscope (SEM) (LEO 1455VP, Zeiss, Oberkochen, Germany) with an electron dispersive X-ray (EDX) analysis unit (EDAX Genesis, EDAX, Mahwah, USA). SEM images were made using a RBSE and a VPSE detector with a resolution of 5 nm. In the SEM images the structure and appearance of the corrosion layer and the implant were evaluated descriptively. EDX analyses performed on regions of special interest resulted in EDX spectra displaying the content of selected elements [17,28].

### 3.8. Mechanical testing

The bending stiffness of the implants was determined in a 3 point bending setup on a universal testing machine (Zwick, Ulm, Germany) after immersion, SEM and weighing. The implants were placed centrally on two supports with a distance of 15 mm between them. The bending punch was located in the middle between the supports. Before the measurement, an initial state was adjusted in which a preload of 2.5 N was applied to the implant. The bending punch was then moved downwards with a constant speed of 1 mm per minute. The degree of the bending was documented by a displacement transducer. A sudden loss of load by more than 10% was defined as the breaking criterion. The measurement stopped as soon as the breaking criterion was reached or the bending punch had moved 5 mm downwards from its initial position [17]. The maximum force (Fmax [N]) and the bending at the point of breakage (ε-Fmax [mm]) were measured. The ε-Fmax is the bending displacement at the moment breakage occurs. It is a measure for the ductility of a material, which reflects the tendency of a material to deform plastically before breakage [48]. To determine the initial stiffness for each material, three implants of each alloy were tested in their initial state.

### 3.9. Statistical Analyses

All statistical tests were done with the programs Microsoft Office Excel®, Version 2003 (Microsoft Cooperation, Redmond, USA) and SPSS® Version 17.0 (SPSS: An IBM Company, Chicago, USA). The results of all measurements were checked for normal distribution. If the results turned out to be normally distributed average values and standard deviations were calculated. For the scores given for the nine 2D images of each animal in the µCT scans mean values were calculated, because their distribution was Gaussian, but for the time-material groups in the µCT as well as the X-ray scoring a median score was calculated and minima and maxima were displayed. When comparing different time points, paired student’s t-tests were performed, while unpaired student’s t-tests were done when comparing the different material groups with one another. In the 3-point bending measurements unpaired student’s t-test were used for comparisons of initial stability and state after explantation, because the implants compared were not the same, since the 3 point bending leads to the destruction of the implants. The level of significance was p ≤ 0.05.

No significant differences were found between data obtained from the µCT scans and the X-rays of the three and six months animals during week two to twelve of AX30 and ZEK100, respectively. Therefore in the repeated measurements, the results of the 3 and 6 months groups of each material were combined to one group.

## 4. Conclusions

In summary, the usage of an *in vivo* µCT is of great advantage in analyzing the *in vivo* degradation of magnesium alloys, because it allows a true quantification of the 3D corrosion over time and qualitative assessment of the corrosion morphology, without interfering with the ongoing experiment. Furthermore, the µCT allows examination of the host reactions at the same time and without additional stress or harm to the laboratory animal. The µCT allows an intraindividual *in vivo* monitoring of the course of the degradation morphology, which will be a key in understanding the degradation of implants of high complexity. No other method can achieve this.

It was shown that in the proximal part of the medullary cavity close to the metaphysis, the corrosion rates of the alloys were much higher than in the diaphysis. Therefore, it can be concluded that the location has a remarkable effect on the rate of corrosion, which is of great importance for possible designs of intramedullar implants made of magnesium alloys and it emphasizes the fact that it is absolutely crucial to test biodegradable magnesium implants *in vivo* and in their target location.

The initial mechanical strength of ZEK100 and its degradation characteristics appear suitable for the application of this magnesium alloy as a material for biodegradable implants in weight bearing bones. Further studies that analyze the long term degradation behavior of ZEK100 are needed. AX30 displays good degradation behavior but it has too little mechanical strength for applications in weight bearing bones. It might be a suitable material for mechanically less demanding applications.

The relation of osseous changes and the extent of corrosion emphasize the need to further assess the biocompatibility of ZEK100 and AX30, especially by histology.

Further research is also needed to fine tune the methods that utilize the µCT to examine the degradation of magnesium implants.
